# Supplementary material for: Loss of microRNA-128 promotes cardiomyocyte proliferation and heart regeneration
Source: Nat Commun. 2018 Feb 16;9:700. doi: 10.1038/s41467-018-03019-z (PMC5816015; doi:10.1038/s41467-018-03019-z)
Supplement: Supplementary file 1 — Supplementary Information [file 41467_2018_3019_MOESM1_ESM.pdf]

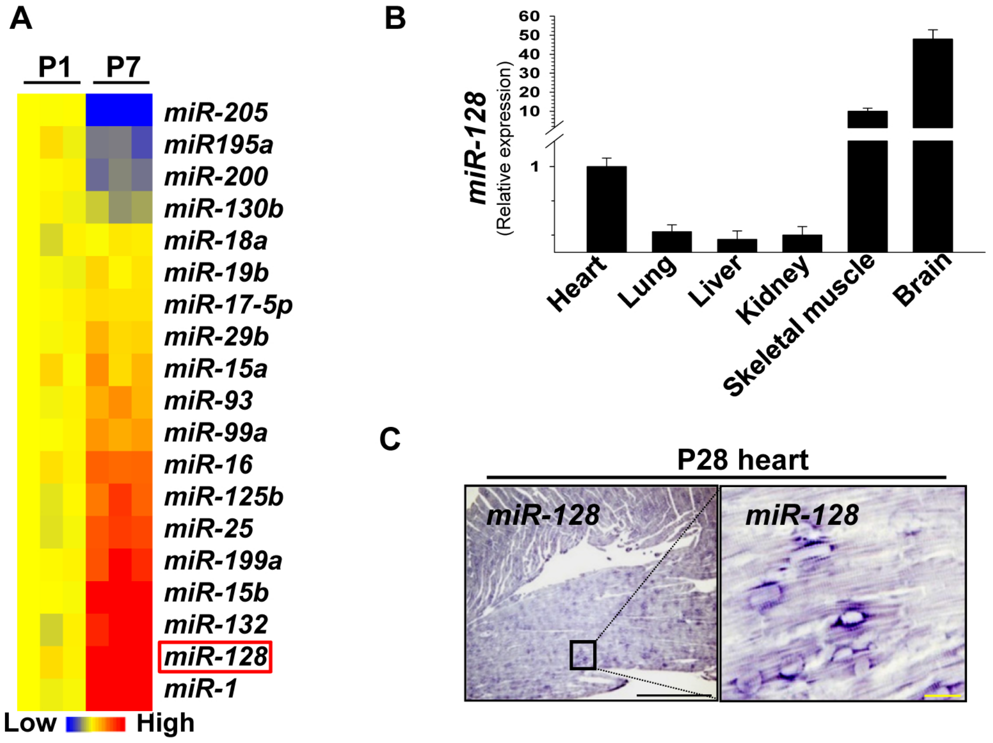

Supplementary Figure 1. Expression of *miR-128* in postnatal hearts

(A) Custom qPCR array of miRNA expression in wild type mouse hearts at postnatal day 1 (P1) and P7. **(B)** Analysis of *miR-128* expression by qPCR in multiple tissues of 8-week old adult mice (n=3). **(C)** Representative images of *miR-128* localization in adult mouse (P28) ventricular cross-sections analyzed by in situ hybridization using digoxigenin-labeled probe. Scale bars, 500 µm (*black*); 25µm (*yellow*).

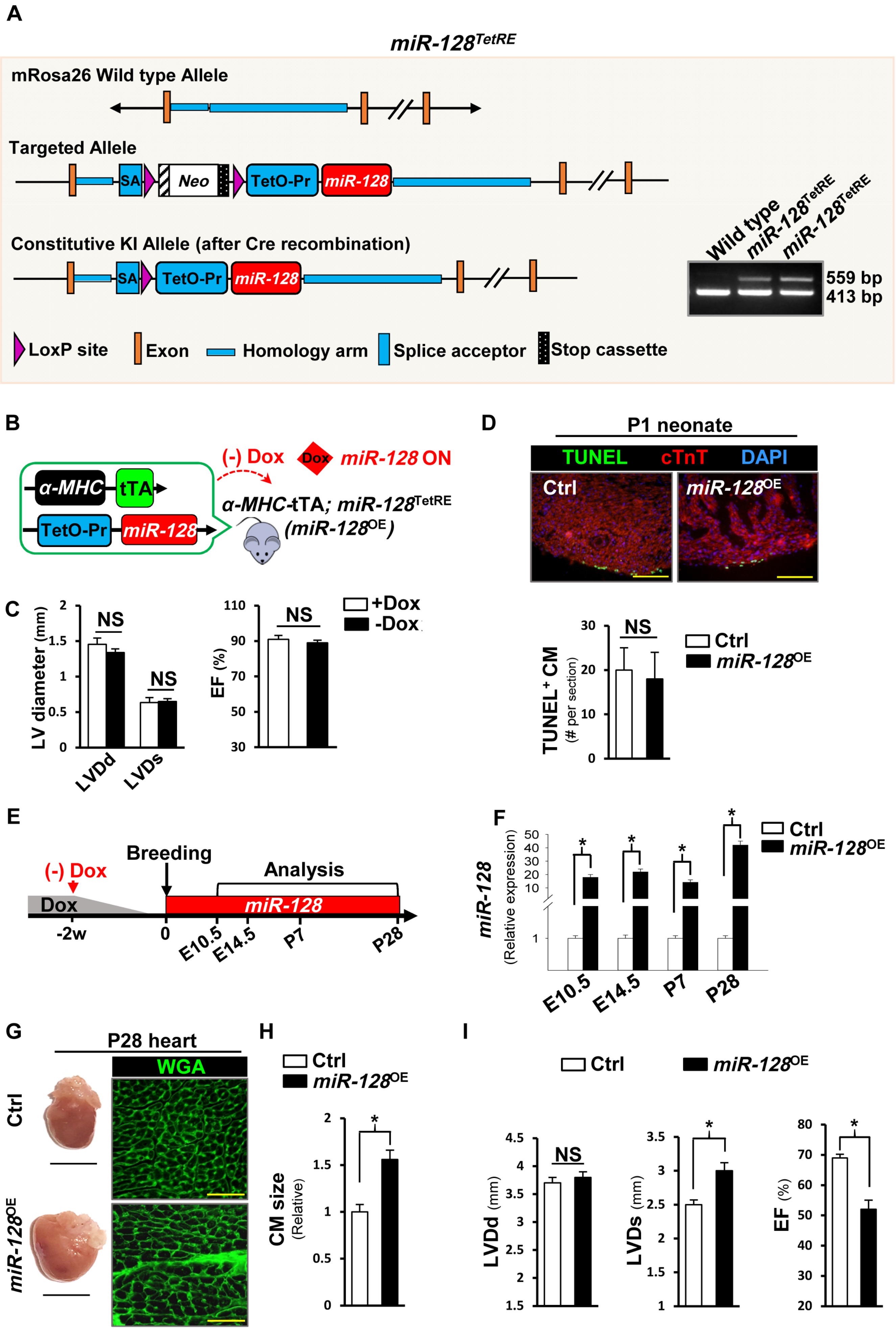
A
miR-128TetRE
mRosa26 Wild type Allele
Targeted Allele
SA
Neo
TetO-Pr
miR-128
Constitutive KI Allele (after Cre recombination)
Wild type
miR-128TetRE
miR-128TetRE
559 bp
413 bp
LoxP site
Exon
Homology arm
Splice acceptor
Stop cassette
B
α-MHC
tTA
(-) Dox
Dox
miR-128 ON
α-MHC-tTA; miR-128TetRE
(miR-128OE)
C
LV diameter (mm)
NS
LVDd
LVDs
EF (%)
+Dox
-Dox
D
P1 neonate
TUNEL
cTnT
DAPI
Ctrl
miR-128OE
TUNEL+ CM (# per section)
E
Breeding
Analysis
-2w
0
E10.5
E14.5
P7
P28
F
miR-128 (Relative expression)
*
G
P28 heart
WGA
H
CM size (Relative)
I
LVDd (mm)
LVDs (mm)

Supplementary Figure 2. Generation of doxycycline-controlled *miR-128* overexpression transgenic mouse

(**A**) The strategy for generating '*miR-128*^{TetRE}' transgenic mouse. TetO-Pr, tetracycline operator promoter. (**B**) Generation of cardiomyocyte-specific *miR-128* overexpressing mice (*miR-128*^{OE}) by breeding *α-MHC*-tTA mice to *miR-128*^{TetRE} mice. Control (Ctrl) mice were designated as *miR-128*^{TetRE} mice, and *miR-128*^{OE} mice were designated as *α-MHC*-tTA; *miR-128*^{TetRE} mice. **(C)** Heart function analyzed by echocardiography in P1 mice as measured by left ventricular end-diastolic diameter (LVDd), LV end-systolic diameter (LVDs), and ejection fraction (EF) (n=6). (**D**) Evaluation of apoptosis in cardiomyocytes (CMs) by TUNEL staining in Ctrl and *miR-128*^{OE} heart sections at P1 (n=3). Scale bars, 100 µm. **(E)** Experimental design to determine the effect of CM-specific overexpression of *miR-128* on heart development. **(F)** Analysis of *miR-128* expression by qPCR in the heart development, including embryonic day 10.5 (E10.5), E14.5, postnatal day 7 (P7), and P28 (n=3). **(G)** Representative images of gross heart morphology (left panel) and Wheat germ agglutinin (WGA) staining (right panel) of heart at P28 (n=5 mice, ~200 CMs/heart). Scale bars, 0.5 cm (*black*); 50 µm (*yellow*). **(H)** Quantification data of CM size analyzed by WGA staining. **(I)** Heart function analyzed by echocardiography in P28 mice as measured by LVDd, LVDs, and EF (n=6). Statistical significance was calculated using Student's *t*-test in **C, D, F, H and I**. Data are represented as means ± SEM. *P <0.05. NS, not significant. Dox, Doxycycline.

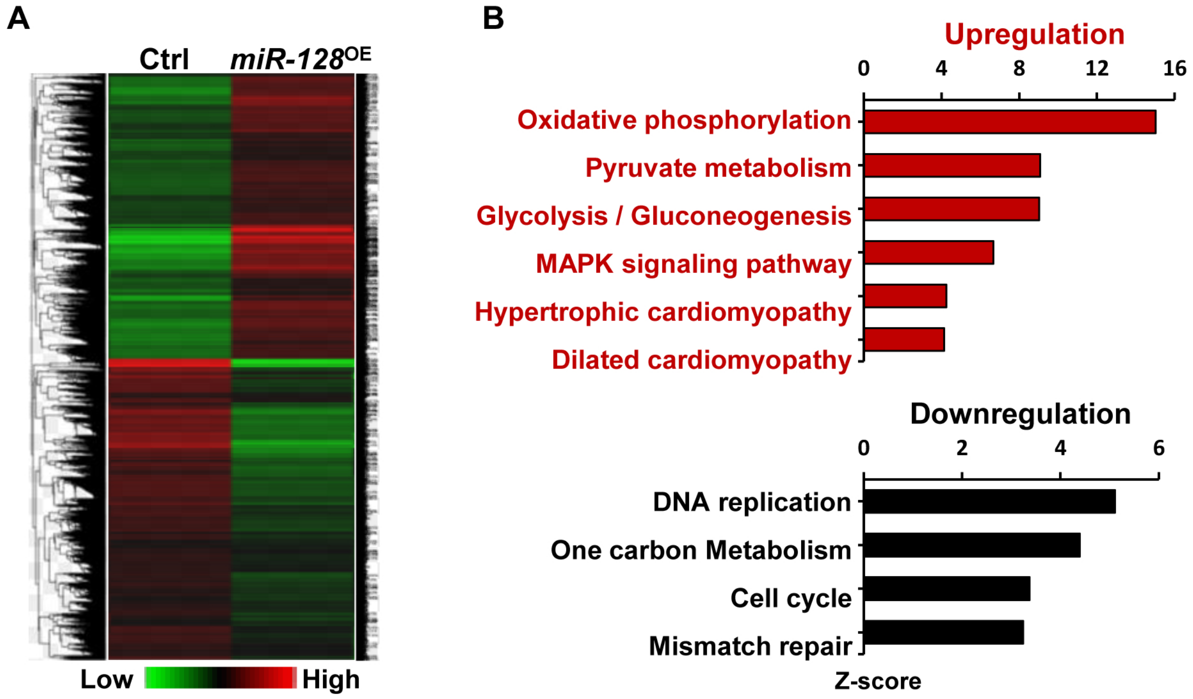

Supplementary Figure 3. Distinct gene-expression profiles of *miR-128*^{OE} hearts

(A) Heat map of the RNA-seq data showing 1,761 upregulated and 1,639 downregulated genes in *miR-128*^{OE} compared to Ctrl hearts at P7. **(B)** KEGG (Kyoto Encyclo-pedia of Genes and Genomes) pathway analysis illustrating a subset of upregulated and downregulated pathways in *miR-128*^{OE} hearts as compared to Ctrl hearts at P7. Control (Ctrl) mice were *miR-128*^{TetRE} mice, and *miR-128*^{OE} mice were *α-MHC*-tTA; *miR-128*^{TetRE} mice.

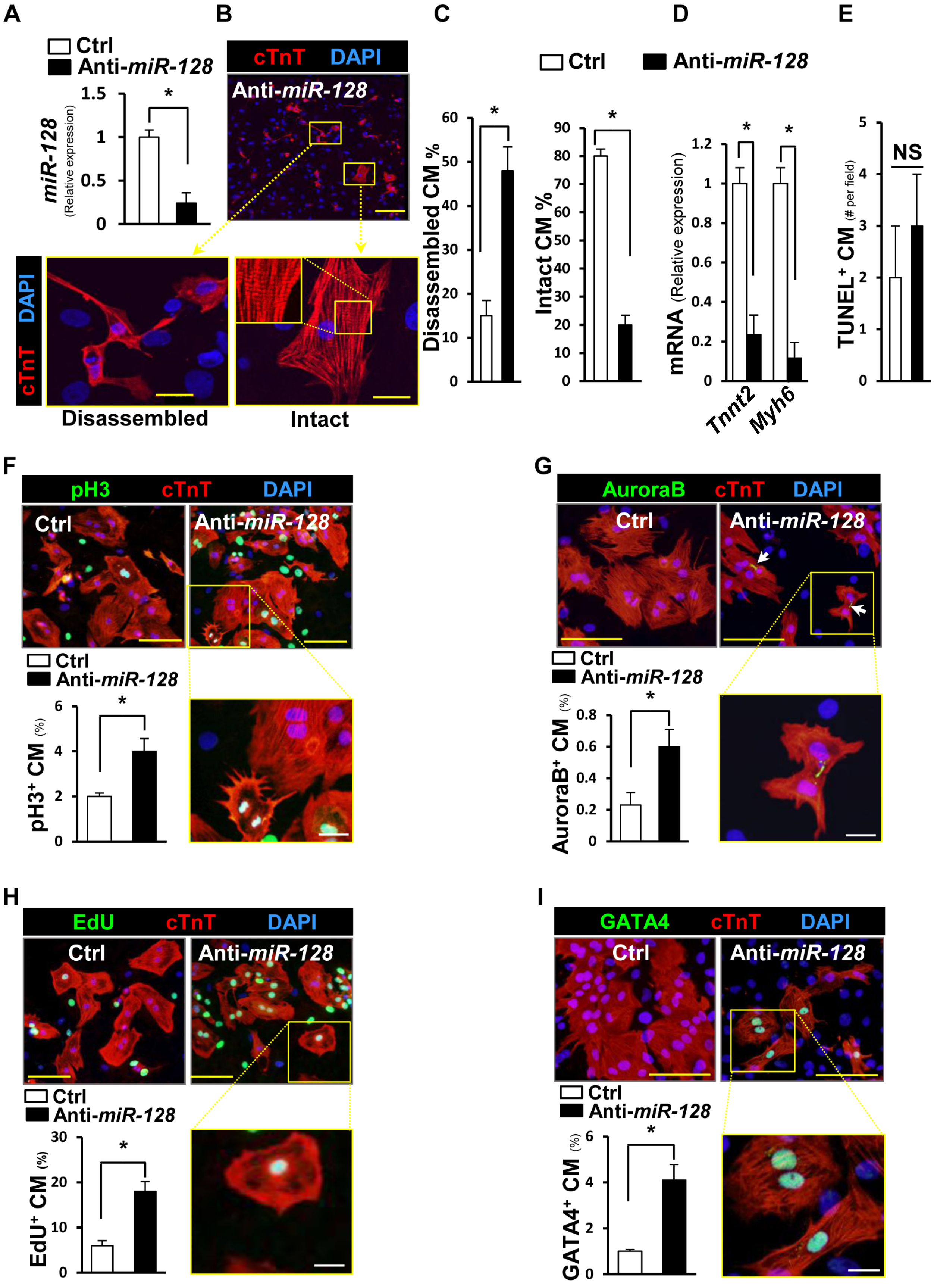

A
Ctrl
Anti-miR-128
miR-128 (Relative expression)
B
cTnT DAPI
Anti-miR-128
Disassembled
Intact
C
Disassembled CM %
Intact CM %
D
mRNA (Relative expression)
Tnnt2
Myh6
E
TUNEL+ CM (# per field)
NS
F
pH3 cTnT DAPI
Ctrl
Anti-miR-128
pH3+ CM (%)
G
AuroraB cTnT DAPI
AuroraB+ CM (%)
H
EdU cTnT DAPI
EdU+ CM (%)
I
GATA4 cTnT DAPI
GATA4+ CM (%)

Supplementary Figure 4. *MiR-128* inhibition promotes CM proliferation in vitro

(A) Analysis of *miR-128* expression by qPCR in neonatal (P1) rat CMs treated with either negative control (Ctrl) or *miR-128* inhibitor (Anti-*miR-128*). **(B)** Representative images for CM sarcomere structure analyzed by cTnT immunofluorescence. Scale bars, 100 µm (upper panel), 20 µm (lower panel). **(C)** Quantification of CM numbers with disassembled sarcomere and intact sarcomere analyzed by cTnT immunostaining (n=5 mice, ~500 CMs/heart). **(D)** qPCR analysis of gene (*Tnnt2* and *Myh6*) expression in CMs**.** **(E)** Quantification of CM apoptosis by TUNEL staining. **(F)** Immunofluorescence staining with pH3 antibody to assess CM proliferation (n=5 mice, ~500 CMs/heart). Scale bars, 50 µm (*yellow*); 10 µm (*white*)**.** **(G)** Immunofluorescence staining of CM proliferation by Aurora B kinase assay (n=5 mice, ~400 CMs/heart).). Scale bars, 50 µm (*yellow*); 10 µm (*white*)**.** **(H)** Immunofluorescence staining by EdU incorporation to assess CM proliferation (n=5 mice, ~500 CMs/heart). Scale bars, 50 µm (*yellow*); 10 µm (*white*)**.** **(I)** Immunofluorescence staining of GATA4 (n=5 mice, ~400 CMs/heart). Scale bars, 50 µm (*yellow*), 10 µm (*white*)**.** Statistical significance was calculated using Student's *t*-test in **A, C, D, E, F, G, H, and I**. Data are represented as means ± SEM (n=3). **P* <0.05. NS, not significant.

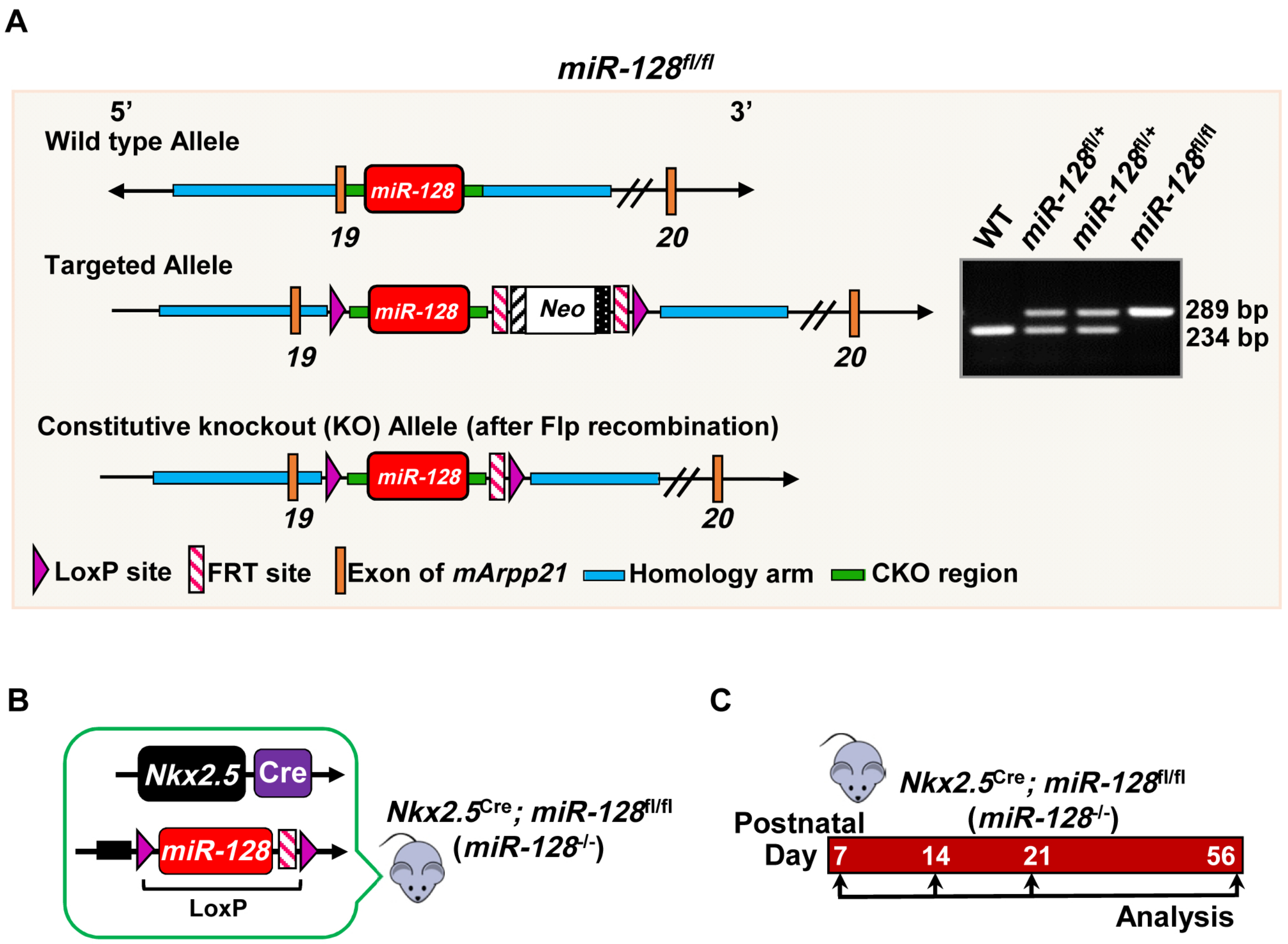

Supplementary Figure 5. Generation of cardiac-specific *miR-128* knockout mouse

(A) The strategy for generating *miR-128*$^{fl/fl}$ mice. The Flp recombinase was used to remove the neomycin gene (Neo) which was flanked by FRT (Flippase Recognition Target sites). **(B)** Generation of cardiac-specific *miR-128* knockout mice were generated by crossing *Nkx2.5*-Cre mice with *miR-128*$^{fl/fl}$ mice (*Nkx2.5*-Cre; miR128$^{fl/fl}$ were designated as *miR-128*$^{-/-}$). Control mice were *miR-128*$^{fl/fl}$ mice. **(C)** A schematic diagram depicting the experimental design in Fig. 3.

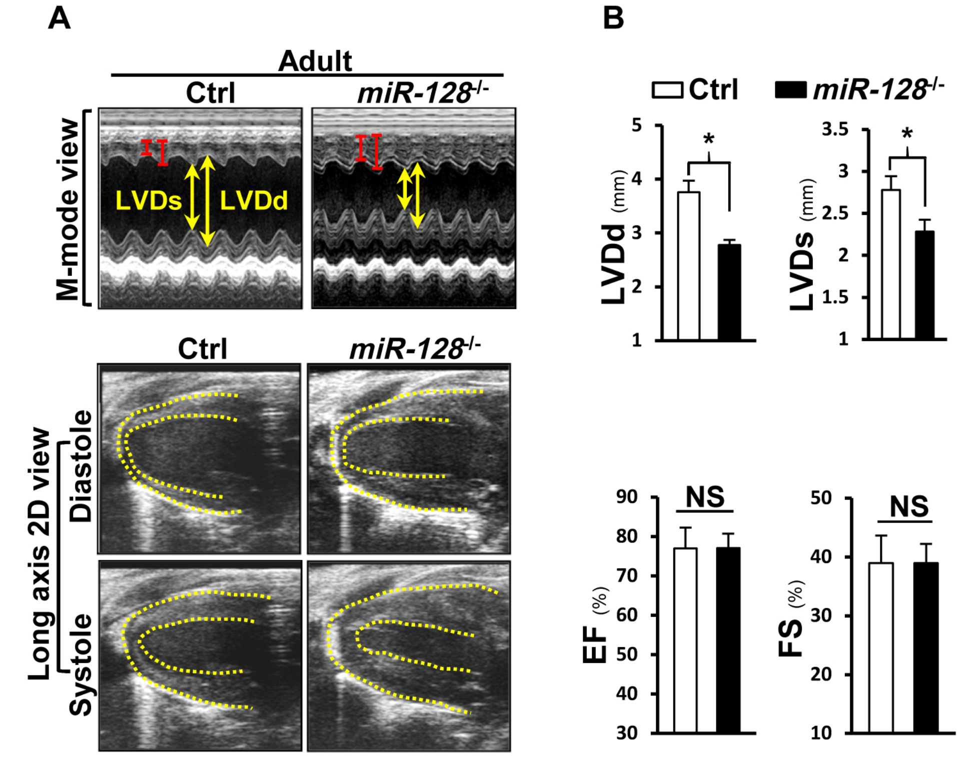

Supplementary Figure 6. Effect of *miR-128* deletion on adult heart function

(A) Heart function of adult mice (8 weeks old) as analyzed by echocardiography. **(B)** Heart function quantified by LVDd, LVDs, EF, and FS. Control (Ctrl) mice were *miR-128*$^{fl/fl}$ mice, *miR-128*$^{-/-}$ mice were *Nkx2.5*-Cre; *miR-128*$^{fl/fl}$. Statistical significance was calculated using Student's *t*-test in **B** (n=5). Data are means ± SEM. *P <0.05. NS, not significant.

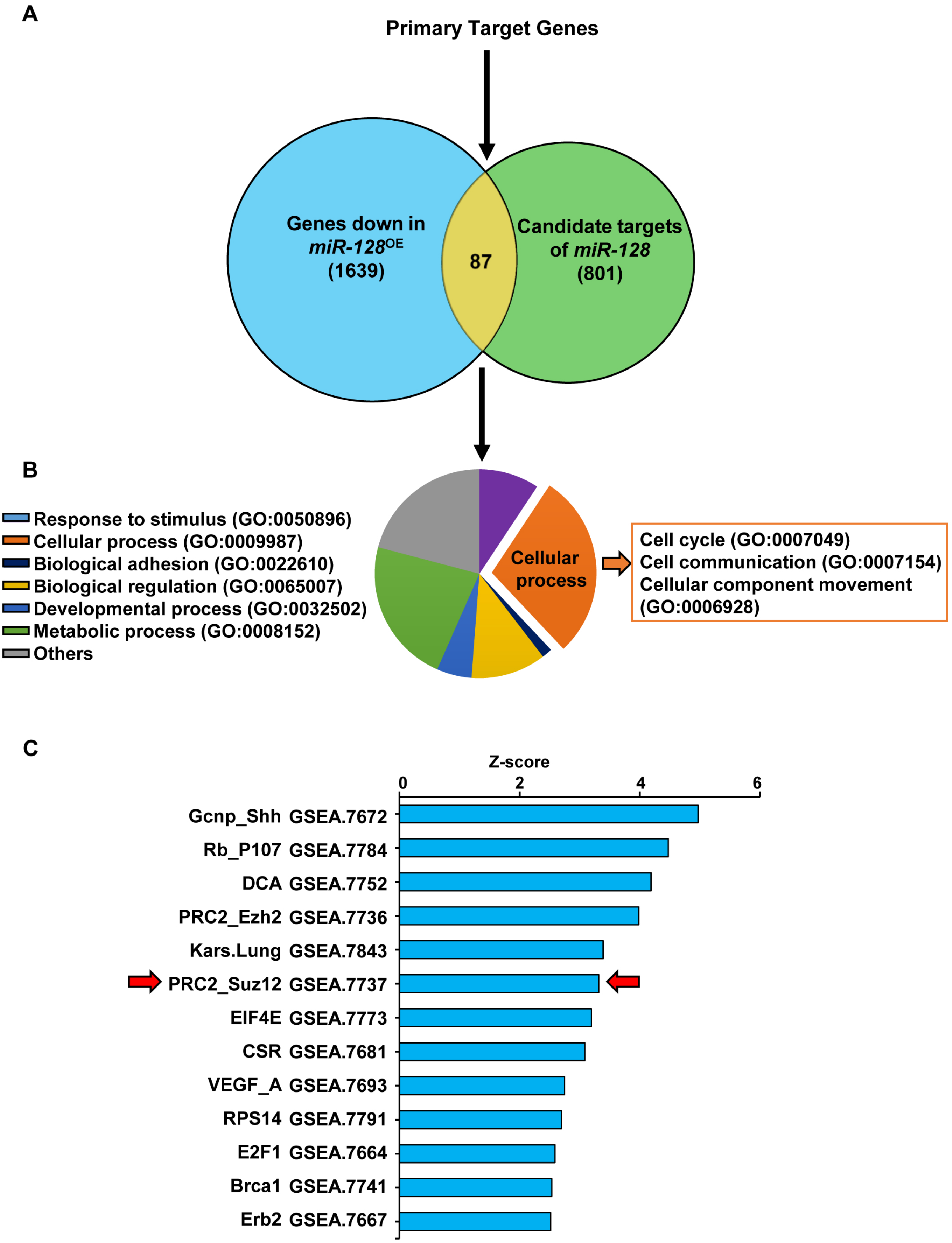
A
Primary Target Genes
Genes down in miR-128OE (1639)
87
Candidate targets of miR-128 (801)
B
Response to stimulus (GO:0050896)
Cellular process (GO:0009987)
Biological adhesion (GO:0022610)
Biological regulation (GO:0065007)
Developmental process (GO:0032502)
Metabolic process (GO:0008152)
Others
Cellular process
Cell cycle (GO:0007049)
Cell communication (GO:0007154)
Cellular component movement (GO:0006928)
C
Z-score
0
2
4
6
Gcnp_Shh GSEA.7672
Rb_P107 GSEA.7784
DCA GSEA.7752
PRC2_Ezh2 GSEA.7736
Kars.Lung GSEA.7843
PRC2_Suz12 GSEA.7737
EIF4E GSEA.7773
CSR GSEA.7681
VEGF_A GSEA.7693
RPS14 GSEA.7791
E2F1 GSEA.7664
Brca1 GSEA.7741
Erb2 GSEA.7667

Supplementary Figure 7. *MiR-128* regulates cell cycle through PRC2-*Suz12* pathway

(A) Venn diagram of genes that were down-regulated in P7 *miR-128*^{OE} hearts (blue) as compared to the Ctrl hearts and predicted candidate target genes of *miR-128* (green). The overlapping 87 genes (yellow) were considered as direct *miR-128* targets. **(B)** Gene Ontology (GO) biological processes identified by PANTHER analysis using the overlapping 87 target genes of *miR-128*. **(C)** Enrichment analysis of down-regulated genes in in *miR-128*^{OE} hearts. Genes down-regulated in TIG3 cells upon knockdown of *Suz12* gene (PRC2_SUZ12_UP.V1_DN) were enriched in *miR-128*^{OE} hearts (red arrow).

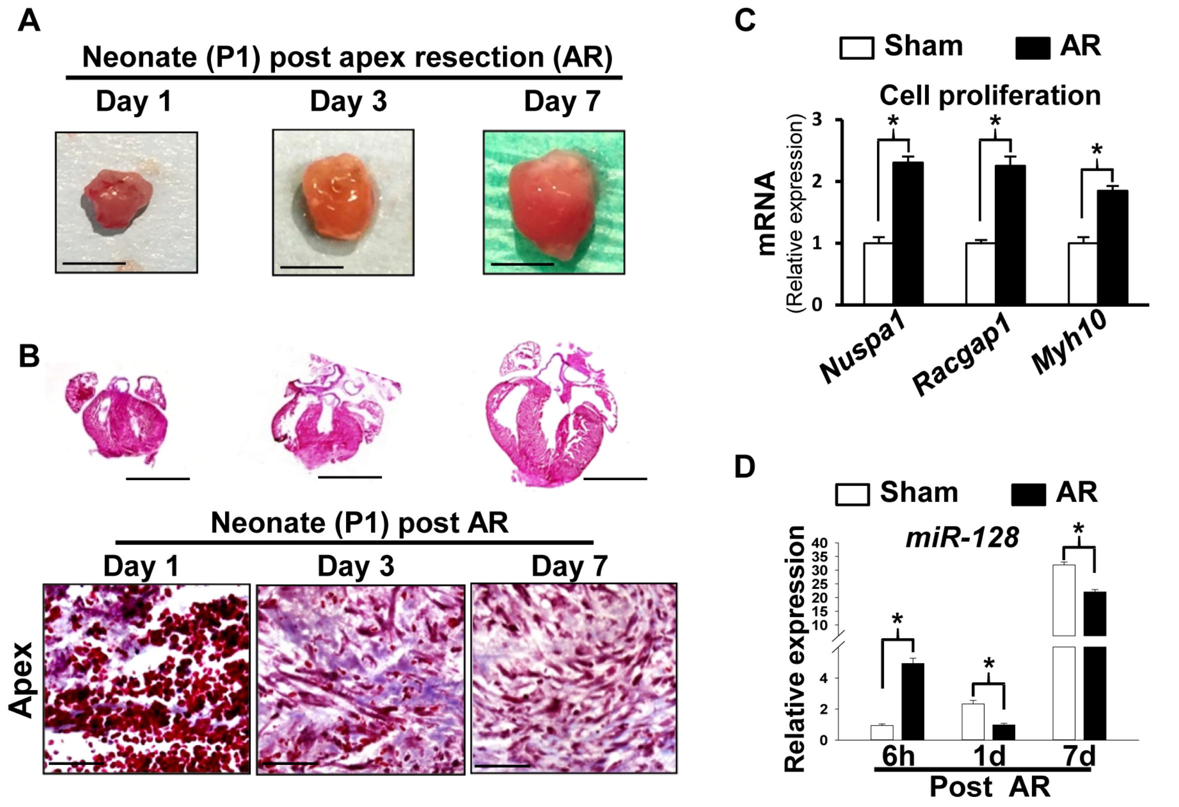

Supplementary Figure 8. Downregulation of *miR-128* in neonatal mice during regeneration after injury

(A) Representative gross (stereomicroscopic) morphology of wild type hearts at day 1, 3, and 7 following apex resection (AR) in P1 mice. Scale bars, 0.25 cm. **(B)** Representative image of Masson's Trichrome staining of hearts at day 1, 3, and 7 following AR in neonatal P1 heart (upper) and higher magnification of resected area (lower). Scale bars, 25 μm. **(C)** qPCR analysis of cell cycle-related gene expression in neonatal hearts at day 7 following AR. **(D)** qPCR analysis of *miR-128* expression in neonatal hearts following AR. Statistical significance was calculated using Student's *t*-test in **C** and **D**. Data are represented as means ± SEM (n=5). *P <0.05.

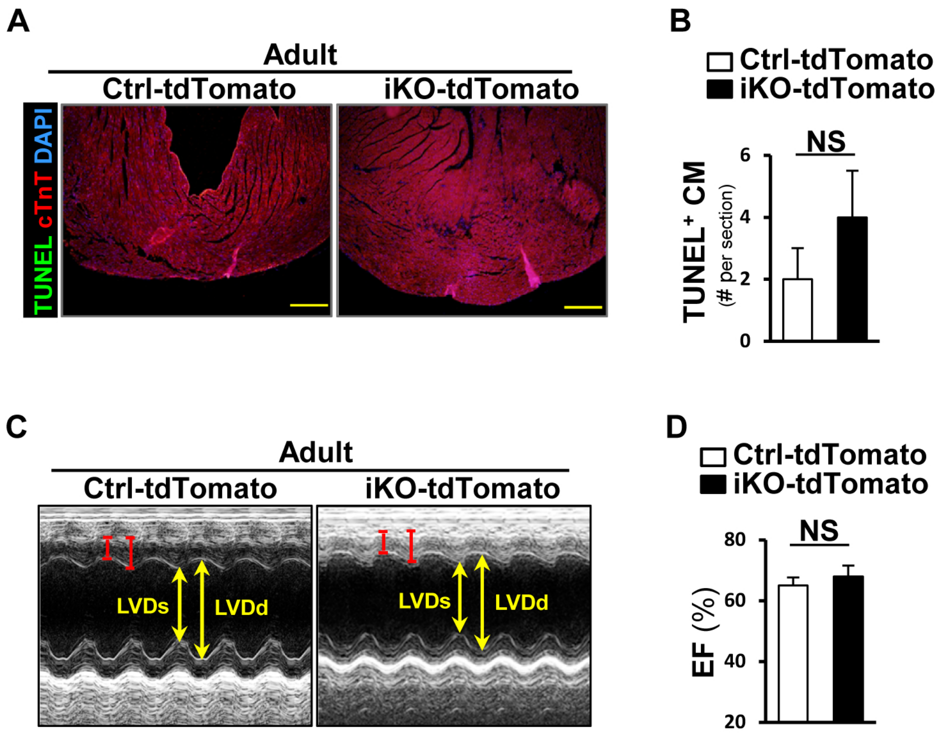

Supplementary Figure 9. Effects of inducible *miR-128* knockout on normal heart

(A-B) CM apoptosis analyzed by TUNEL staining on 6 week old control mice (*α-MHC*^{MerCreMer}; RosatdTomato, designated as Ctrl-tdTomato) and inducible *miR-128* knockout mice (*α-MHC*^{MerCreMer}; *miR-128*$^{\text{fl/fl}}$; RosatdTomato, designated as iKO-tdTomato). Scale bars, 200 µm. **(C-D)** Heart function analyzed by echocardiography and quantified by EF. Statistical significance was calculated using Student's *t*-test in **B** and **D** (n=5). Data are means ± SEM. *P <0.05. NS, not significant.

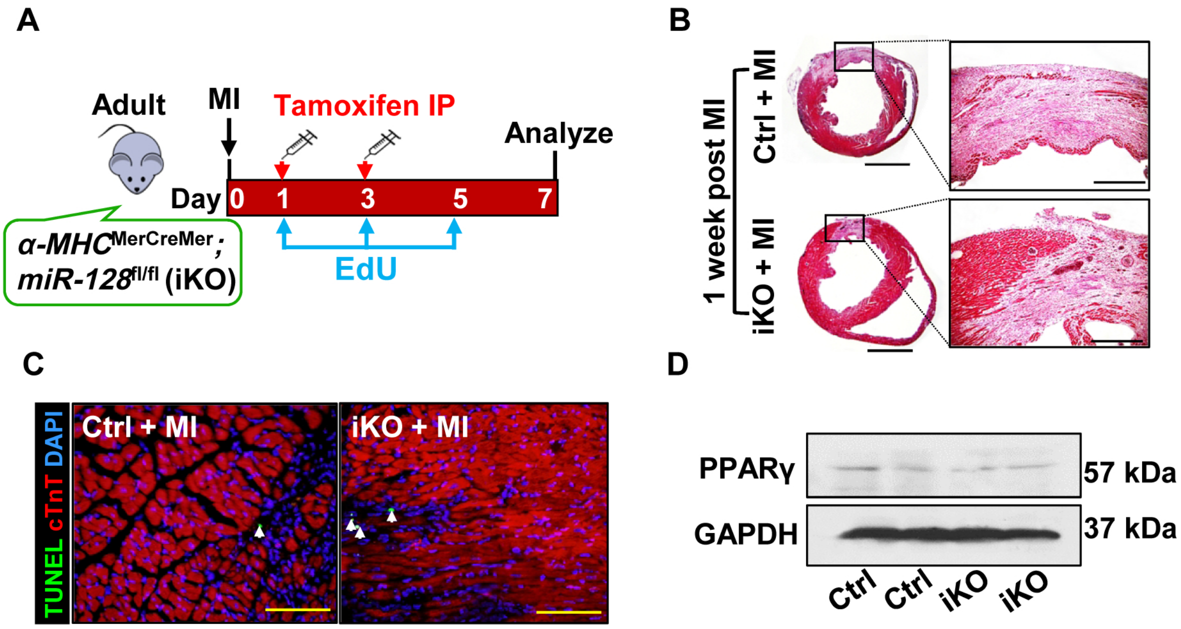

Supplementary Figure 10. *MiR-128* deletion promotes cardiac regeneration in the adult heart after MI

(A) A schematic diagram depicting the experimental design using adult transgenic mice (12 week old). **(B)** Masson's Trichrome staining of control (*miR-128*$^{fl/fl}$, designated as Ctrl) and *miR-128* knockout (*α-MHC*^{MerCreMer}; *miR-128*$^{fl/fl}$, designated as iKO) hearts at 1 week post tamoxifen (TAM) administration. Scale bars, 1 mm (*left panel*); 200 μm (*right panel*). **(C)** Evaluation of CM apoptosis by TUNEL assay. Scale bars, 50 μm. **(D)** Western blot assay of peroxisome proliferator-activated receptor gamma (PPARγ) expression at 1 week post TAM administration.

Fig. 4B

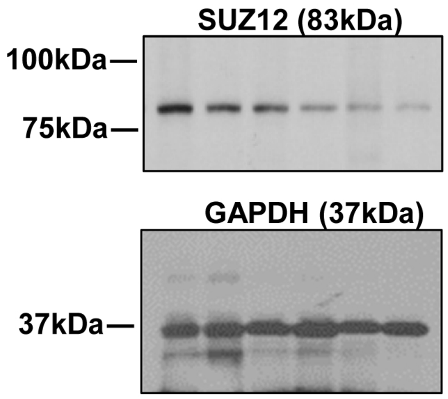

Fig. 4D

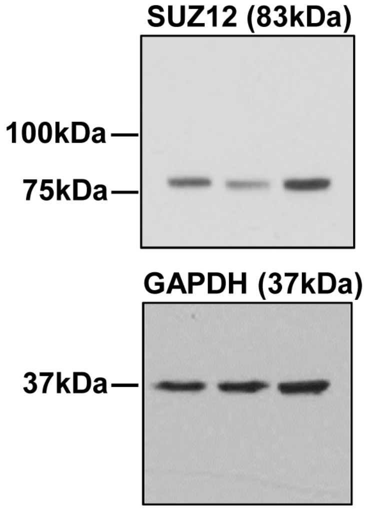

Fig. 4G

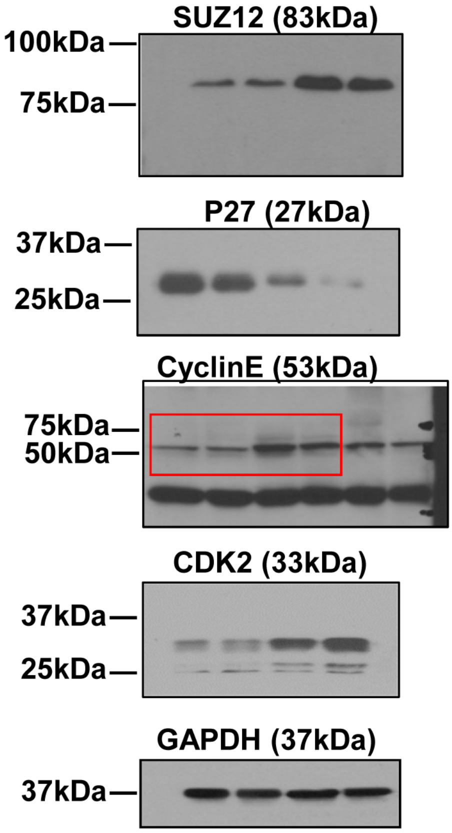

Fig. 5G

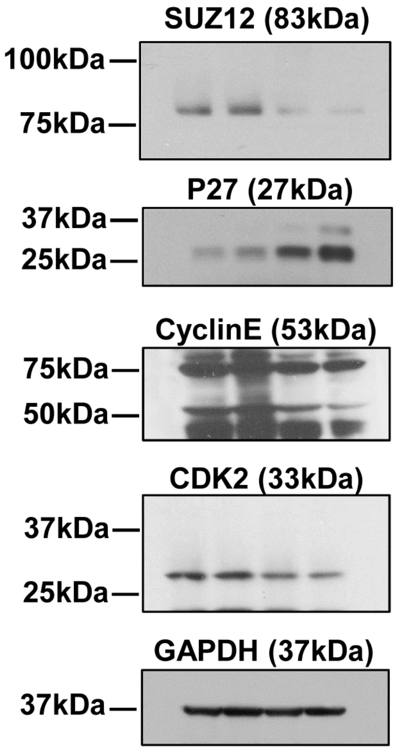

Fig. 8B

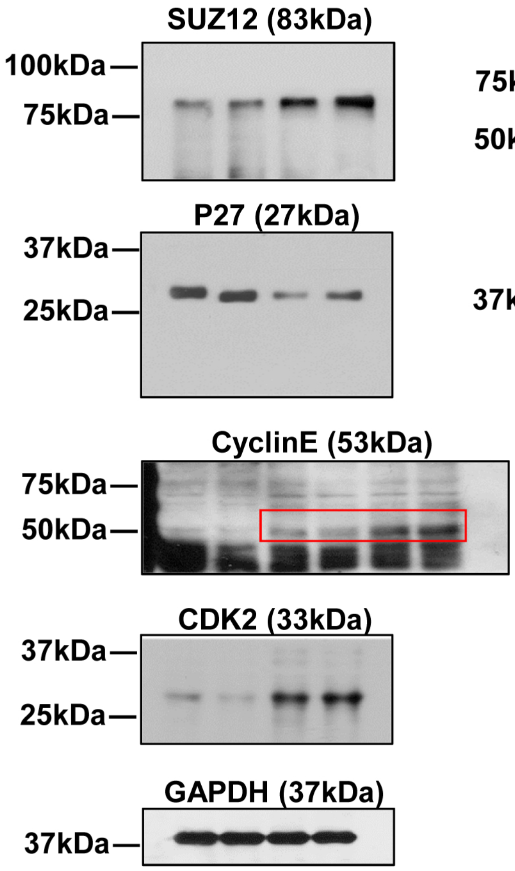

Supplementary Fig. 10D

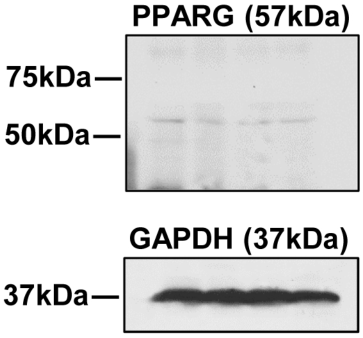

Supplementary Figure 11. Uncropped images of immunoblots with molecular weight markers

Uncropped images of the immunoblots with molecular weight markers (left side) displayed in the main and supplementary figures of the manuscript. The red boxes show the cropped blots of the identified proteins.

Supplementary Table 1. PCR primers and oligonucleotides

**Target: Sequences (5'-3')**
**RT-qPCR:**<br><br>*miR-128*: CGTCACAGTGAACCGGTCTCT;<br><br>miRNA universal reverse primer: provided by miScript PCR Starter Kit;<br><br>U6-F: CTCGCTTCGGCAGCACA;<br><br>U6-R: AACGCTTCACGAATTTGCGT;<br><br>*Racgap1*-F: CAGATCCAGTGACAATGTTCCA;<br><br>*Racgap1*-R: TCCACCATCATGAACTGATTCC;<br><br>*Nusap1*-F: GAGGAGGAAGAAGCACAAGAC;<br><br>*Nusap1*-R: CTACTATCAGTTCCTTTCATCTCCAA;<br><br>*Myh10*-F: GGAATTCGAGAGGCAGAACAA;<br><br>*Myh10*-R: AAGGCTCGCTTGGATTTCTC;<br><br>*Nppa*-F: CTGAGGTGCCTCCCTGGAC;<br><br>*Nppa*-R: ACTCTGGGCTCCAATCCTGTC;<br><br>*Nppb*-F: AAGGACCAAGGCCTCACAAA;<br><br>*Nppb*-R: GCCAGGAGGTCTTCCTACAAC;<br><br>*Myh6*-F: GGACGCCCAGATGGCTGACT;<br><br>*Myh6*-R: CCTTGTCATCAGGCACGAAGCAC;<br><br>*Myh7*-F: GTTTGTCAAGGCCAAGATCGTGT;<br><br>*Myh7*-R: AGCATGGCCATGTCCTCGAT;<br><br>*Tnnt2*-F: TTCATGCCCAACTTGGTGCC;<br><br>*Tnnt2*-R: CTCTTCAGCCAGGCGGTTCT;
**ChIP-qPCR:**<br><br>(SUZ12, EZH2 and H3K27me3) *p27*-promoter-F: GAGATCCTACGGTGGAAGCG;

(SUZ12, EZH2 and H3K27me3) *p27*-promoter-R: CTTAGCTGGGGTGCGGAATC;
**Genotyping of *miR-128* knockin**<br>Targeted transgenic allele-F: TGAGCCAGACCTCCATCGC<br>Targeted transgenic allele-R: AGCTCGGTACCATTAATCG<br>Wild type allele-F: ACTCCAAGGCCACTTATCACC<br>Wild type allele-R: ATTGTTACCAACTGGGACGACA
**Genotyping of *miR-128* knockout**<br>Targeted transgenic allele-F: GCCCTAATTTGATCATCAGAACC<br>Targeted transgenic allele-R: CATTGTTGTAGCCACACCCC<br>Wild type allele-F: TCATAGCTGTACTTACTGGATG<br>Wild type allele-R: CACTTGGGCTTGGAAGATAG
**Cloning of *Suz12* 3'UTR**<br>Wild type F: CCGCTCGAGGTTTCCTTTGTGACATCCTTCTTGA<br>Wild type R:<br>AAGGAAAAAAGCGGCCGCATACCAGTATTTACAGCAGCATTGA<br>Mutant F: cacGTTGAATAACATCACCTCAATTTTTTATTATC<br>Mutant R: tctgCTTGGAGCTGGAATAAAAAG
